# Sonographic cervical motion tenderness: A sign found in a patientÂ with pelvic inflammatory disease

**DOI:** 10.1186/2036-7902-4-20

**Published:** 2012-09-18

**Authors:** Resa E Lewiss, Turandot Saul, Katja Goldflam

**Affiliations:** 1 Department of Emergency Medicine, St. Luke's/Roosevelt Hospital Center, 1111 Amsterdam Avenue, New York, NY, 10025, USA; 2 Department of Emergency Medicine, Yale-New Haven Hospital, 464 Congress Avenue, Suite 260, New Haven, CT, 06519, USA

**Keywords:** Pelvic inflammatory disease, PID, Cervical motion tenderness, Sonographic CMT, Bedside ultrasound

## Abstract

No single historical, physical, laboratory, or imaging finding is both sensitive and specific for the diagnosis of pelvic inflammatory disease (PID). Cervical motion tenderness (CMT), when present, is classically found on bimanual examination of the cervix and uterus. CMT is often associated with PID but can be present in other disease entities. We present a case report of a patient who was ultimately diagnosed with acute PID. The evaluating physician performed a trans-vaginal bedside ultrasound, and the operator appreciated ‘sonographic CMT’. In cases where the physical examination is equivocal or in patients where the exact location of tenderness is difficult to discern, performing a trans-vaginal bedside ultrasound examination can increase the physician's confidence that CMT is present as the cervix is being directly visualized as pressure is applied with the probe. Bedside ultrasound and specifically sonographic CMT may prove useful in diagnosing PID in patients with equivocal or unclear physical examination findings.

## Background

No single historical, physical, laboratory, or imaging finding is both sensitive and specific for the diagnosis of pelvic inflammatory disease (PID). According to the 1991 Center for Disease Control guidelines for the prevention and management of PID, treatment should be initiated on the basis of the following minimum clinical criteria for pelvic inflammation: lower abdominal tenderness, bilateral adnexal tenderness, or cervical motion tenderness (CMT). Additional criteria for the diagnosis include oral temperature >38.3°C, elevated erythrocyte sedimentation rate and/or C-reactive protein, culture or non-culture evidence of cervical infection with *Neisseria gonorrhoeae* or *Chlamydia trachomatis*, or histo-pathological evidence on endometrial biopsy and/or laparoscopy [1].

Pain upon movement of the cervix with the health care provider's gloved fingers is suggestive of an inflammatory process of the pelvic organs. CMT, when present, is classically found on bimanual examination of the cervix and uterus. While CMT is often associated with pelvic inflammatory disease, it can be present in other disease entities such as ectopic pregnancy, endometriosis, ovarian torsion, appendicitis, and perforated abdominal viscus.

PID is not a singular disease entity but describes a spectrum of disease. It is an upper genital tract infection, which may affect the uterus, fallopian tubes, ovaries, and peritoneum. PID can begin as cervicitis, progress to endometritis, followed by involvement of the fallopian tubes as pyosalpinx, and ultimately involve the ovary as a tubo-ovarian abscess (TOA). The two most common causative pathogens are *N. gonorrhoeae* and *C. trachomatis* although the infection is often polymicrobial [2].

The American Institute of Ultrasound in Medicine practice guidelines for the performance of pelvic sonography lists pelvic pain, an abnormal or technically limited pelvic examination, or signs or symptoms of pelvic infection, each as indications for applying this modality [3].

Bedside emergency ultrasound (BUS) is an important diagnostic tool for the emergency physician (EP). Historically, BUS focused on the evaluation of women presenting with pelvic pain or bleeding focused on patients in their first trimester of pregnancy. The gynecological literature and, increasingly, the emergency medicine literature support the utility of BUS in the evaluation of pelvic complaints in non-pregnant women.

We present a case report of a patient who was ultimately diagnosed with acute PID and an associated tubo-ovarian abscess. The evaluating physician performed a trans-vaginal BUS, and the operator appreciated ‘sonographic CMT’ in this patient.

## Case presentation

### Case report

A 25-year-old HIV-negative female presented to the emergency department with constant sharp left lower quadrant pain for 1 day accompanied by nausea and three episodes of vomiting. Her last menstrual period was 3 weeks prior to presentation. Although the patient had received treatment for PID 2 weeks prior to this visit, her male sexual partner had not been treated for any sexually transmitted infections. She denied diarrhea, fever, urinary symptoms, vaginal bleeding, or discharge. At triage, her temperature was 101.0°F, blood pressure was 100/60 mmHg, heart rate was 110 beats/min, respiratory rate was 20/min, and oxygen saturation was 96% on room air. The patient had no surgical history, and sexual history was significant for multiple male partners without consistent use of barrier protection.

On physical examination, the lungs were clear, the heart sounds were fast and regular, and the abdomen was soft with left lower quadrant tenderness without rebound or guarding. Pelvic examination was difficult to interpret as the patient was in a significant degree of discomfort. On speculum examination, there was no blood in the vaginal vault, and there was a small amount of clear discharge from the cervical os. On bimanual examination, the patient had tenderness of the cervical area, the uterus, and the bilateral adnexae, left greater than right. It was unclear if distinct CMT was present as the patient was uncomfortable with the entire bimanual examination. A point-of-care test of the urine was negative for pregnancy and infection.

**Figure 1:**
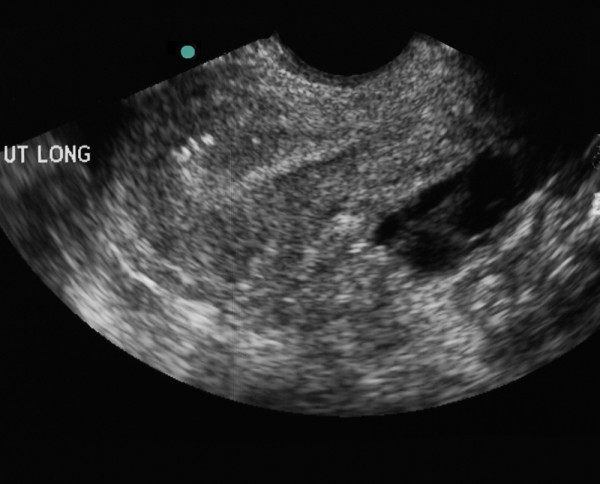
** Sagittal view of the uterus on trans-vaginal ultrasound.** A bulky heterogeneous anteverted uterus is seen. The striated pattern suggests inflammation and infection.

**Figure 2:**
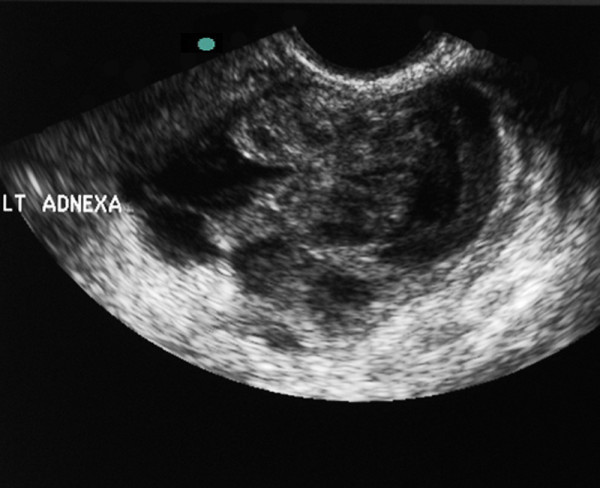
** Coronal view of left tubo-ovarian complex.** A more organized heterogeneous collection consistent with an abscess.

A BUS was performed. The patient tolerated the probe insertion but had a significant amount of pain when the probe abutted the cervix (Figure 1). With pressure applied to the probe to directly visualize the cervix, the patient felt a focal exacerbation of her pain, which the EP recognized, clarified, and confirmed as CMT. A coronal view of the left adnexal region demonstrated a complex heterogeneous collection thought to represent an abscess (Figure 2).

The gynecology service was consulted, and an ultrasound in the department of radiology was obtained to further evaluate the adnexae and document blood flow to the ovaries. The left adnexa was found to be an enlarged tubo-ovarian complex. It was estimated to measure 8 cm in diameter with multiple irregular tubo-ovarian abscesses. The ovaries demonstrated bilateral blood flow. There was a small amount of free fluid in the posterior cul-de-sac.

Laboratory studies showed a WBC count of 15.0. The patient was started on intravenous antibiotics and admitted to the gynecology service. Microbiology result of the cervical culture was positive for *N. gonorrhea.*

## Discussion

BUS is increasingly being utilized to evaluate pelvic pain in the non-pregnant female. We describe the finding of sonographic CMT in a patient who presented to the ED with pelvic pain and a difficult to localize physical examination. The patient was ultimately diagnosed with PID and a TOA.

The presence of CMT is an important finding during the bimanual examination and is suggestive of certain pelvic inflammatory processes. This finding may heighten suspicion of one disease entity over another in these patients and help to prioritize the differential diagnosis. Trans-vaginal ultrasound is performed using a high-frequency (5 to 8 MHz) endocavitary probe. The probe is inserted into the vaginal canal, providing excellent resolution of the cervix, uterus, and ovaries. During the ultrasound examination, the probe comes into contact with the cervix, and cervical motion results as the operator scans through the entire uterus in sagittal and coronal planes and then laterally to visualize each ovary. The cervix can be directly visualized and the practitioner can be clear that tenderness elicited is from cervical pressure and motion.

In 1997, Boardman et. al. prospectively enrolled 50 women with classic and nonclassic presentations of PID to note the trans-vaginal sonographic presence of cul-de-sac fluid for cul-de-sac fluid, size and number of ovarian cysts, presence of fallopian or fluid-filled tubes, and presence of an adnexal mass or TOA. Fallopian tube identification during trans-vaginal sonography was considered an abnormal finding in addition to multicystic ovaries. They noted a high positive predictive value in identifying a fallopian tube, tubal fluid, or a TOA [4].

In their prospective convenience sample study, Tayal et al. hypothesized that the use of trans-vaginal ultrasound by EPs would change diagnostic decision making in the evaluation of adult non-pregnant female patients with right lower quadrant pain. They reported that the sonologists felt that the sonographic examination complimented the physical examination, and of 40 patients enrolled, 12 had gynecological findings to explain the pain. The authors suggested that BUS could change the physician's diagnostic decision making in a certain cohort. The authors did not comment upon the evaluation of pain caused by the probe itself [5].

In another study, Tayal et al. discussed the physician's examination confidence and the role of the sonographic bimanual examination (SBME) in the pelvic examination of adult non-pregnant women with lower abdominal conditions. SBME was compared to a traditional digital bimanual examination (DBME). Cervical motion, uterine, and ovarian tenderness were evaluated as a part of this study. Of the 30 women enrolled, the SBME provided improved confidence in overall and key aspects of the pelvic examination across body mass index classes as compared to the DBME. Physician confidence in the examination was higher for the SBMEs for uterine and ovarian tenderness, but not for cervical motion or retrovaginal tenderness [6].

Adhikari et al. asserted the role of BUS in differentiating PID from TOA. In their retrospective review, all patients had lower abdominal tenderness, 45% (9 of 20) had CMT on physical examination, and only 15% (3 of 20) had an elevated WBC count. Sonographically, a majority (70%) of patients presenting with a TOA had a complex adnexal mass, a quarter had echogenic fluid in the cul-de-sac, and a small portion (15%) had a pyosalpinx [7].

Pelvic ultrasound has a sensitivity of 93% and a specificity of 98% for the diagnosis of TOA [8]. The inflammatory process makes borders between structures difficult to identify, and the sonographic finding of a complex mass may be referred to as the tubo-ovarian complex [9]. If left untreated, PID can lead to tubal scarring, infertility, ectopic pregnancy, and chronic pelvic pain [10].

## Conclusion

Bedside ultrasound and, specifically, sonographic CMT may prove useful in diagnosing signs of pelvic inflammatory disease in patients with equivocal or unclear physical examination findings.

## Consent

Written informed consent was obtained from the patient for publication of this case report and accompanying images. A copy of the written consent is available for review by the Editor-in-Chief of this journal.

## Competing interests

The authors declare that they have no competing interests.

## Authors' contributions

RL, TS, and KG participated in the recruitment of patient data and literature review. RL, TS, and KG participated in writing specific sections and in drafting the complete manuscript. RL, TS, and KG read, edited, and participated in revising the manuscript. All authors read and approved the final manuscript.
